# Food insecurity and health outcomes during the coronavirus pandemic in South Africa: a longitudinal study

**DOI:** 10.1186/s13561-022-00375-x

**Published:** 2022-06-20

**Authors:** Chijioke O. Nwosu, Umakrishnan Kollamparambil, Adeola Oyenubi

**Affiliations:** 1 Department of Economics and Finance, University of Free State University of the Free State, Bloemfontein, 9301 South Africa; 2 School of Economics & Finance, University of the Witwatersrand, Johannesburg, South Africa

**Keywords:** Food insecurity, Hunger, Health, COVID-19, South Africa

## Abstract

**Background:**

Given that South Africa experienced significant food insecurity even before the COVID-19 pandemic, it is not surprising that the pandemic would result in even greater food insecurity in the country. This paper provides additional evidence on the relationship between food insecurity and health.

**Methods:**

Data came from the National Income Dynamics Study-Coronavirus Rapid Mobile Survey, a longitudinal survey of adult South Africans. Health was a self-reported indicator of general health, while food insecurity was measured by household hunger, the frequency of household hunger, and households running out of money to buy food. We performed descriptive and econometric analyses.

**Results:**

Food insecurity has remained high even in the face of greater re-opening of the economy. Moreover, among hunger-affected households, between a quarter and a third struggled with hunger almost daily or daily. Belonging to a hunger-affected household was associated with a 7-percentage point higher probability of worse health compared to not experiencing hunger. Compared to being unaffected by hunger, being hungry everyday was associated with a 15-percentage point higher probability of worse health in wave 1, an effect that became statistically insignificant by wave 4.

**Conclusions:**

These results show the enormity of the hunger problem in South Africa and its adverse effects on health. In the face of economic uncertainty and the removal of COVID-19 palliatives like the grant top-ups, we enjoin policy makers to protect the vulnerable from food insecurity by continuing the implementation of anti-hunger policies and other measures that enhance food security in the country.

## Background

A substantial part of the global population suffered from food insecurity even before the current 2019 coronavirus disease (COVID-19) pandemic. The Food and Agricultural Organization (FAO) defines food security as a situation where all people, at all times, have physical, social and economic access to sufficient, safe, and nutritious food to meet their dietary needs and food preferences for a healthy and active life [1]. According to the FAO’s The State of Food Security and Nutrition in the World Report 2020, almost 690 million people (8.9% of global population) were undernourished in 2019, with the figure expected to exceed 840 million by 2030. Unfortunately, Africa bears a disproportionate share of global undernutrition, with 19% of the continent (more than 250 million people) being undernourished in 2019.^1^

While the full extent of the effect of COVID-19 on food insecurity is not yet known, the pandemic has had a devastating impact on food security globally. This is partly due to domestic food price inflation owing to supply chain disruptions caused by COVID-19 social distancing regulations [2]. Other pandemic control measures such as border restrictions and lockdowns slowed harvests in some countries, leaving seasonal workers without livelihoods while militating against food transport to markets [3].

Like every other country, South Africa has been severely affected by COVID-19. While the country already suffered high levels of food insecurity before the pandemic, there is ample evidence that this situation has likely worsened during the pandemic. Approximately 16% of households reported inadequate food access in 2017, with 5.5% of households describing their access to food as severely inadequate. Moreover, about 11% of households reported vulnerability to hunger [4]. However, the situation worsened considerably during the pandemic. For instance, about 47% of the adult population reported that their households ran out of money to purchase food around April 2020 (a period characterized by the strictest lockdown restrictions in the country) [5]. Also, in May/June 2020, 23% reported that someone in their household went to bed hungry over the past 7 days due to a lack of food. While this figure declined to 16% in July/August, it deteriorated to 18% in November/December 2020, with the worsening of hunger likely driven in part by the removal of government grant top-ups earlier introduced as part of the country’s pandemic response measures [6]. (As part of pandemic palliative measures, the government topped up the child support grant with ZAR300 (USD20) per child in May 2020 and later changed it to ZAR500 (USD33.33) per caregiver per month from June-October 2020. Other grants received a ZAR250 (USD16.67) top-up per month for six months also ending in October 2020 [7]). A November–December 2020 survey by IPSOS revealed that the hunger problem was widespread across the nine provinces, with Kwazulu-Natal (the second most populous province) and the Eastern Cape (one of the poorest provinces) most affected. These provinces reported 58% and 56% hunger prevalence respectively, while the least affected province (Mpumalanga) reported a prevalence of 29%.^2^

The above situation is not very surprising since South Africa is one of the countries most affected by the pandemic in sub-Saharan Africa (SSA). In addition, the country has implemented one of the most robust pandemic response measures globally, consisting of varying levels of lockdown restrictions from March 2020, ranging from level 5 (the most stringent) to level 1 (least restrictive) [8]. These restrictions resulted in several adverse consequences, not least the placing of an already weak labour market under severe stress and partly due to a significant fall in demand which resulted in massive outright job losses and furloughs especially at the beginning of the pandemic [9, 10]. While the government introduced some social assistance programmes like the top-up of existing government grants and the establishment of a Special COVID-19 Relief of Distress grant worth 350 Rands (about USD23) per month for an initial period of six months (eventually extended to 9 months) payable to unemployed South Africans, these measures were clearly inadequate to compensate for job losses and already high unemployment rates. As noted elsewhere, about 3 million South Africans lost their jobs between February and April 2020 [11]. While these figures partly recovered over time, recent figures indicate that the unemployment rate, already at the worryingly high level of 29% in the last quarter of 2019, reached a high of 32.5% in the final quarter of 2020 [12].

This situation would no doubt have exacerbated an already worrying situation. Prior to the pandemic, a number of indicators, like stunting and hunger, suggested that food insecurity in South Africa was too high for an upper middle-income country (Jacobs P, Nwosu CO, Parker W-a, Nyamwanza A, Mabharwana N, Babalola M, et al: A comprehensive status report on population, food and nutrition security and sustainable development in South Africa, unpublished), [13]. Unfortunately, the pandemic has worsened the situation, with many households experiencing hunger and acute lack of resources to purchase food [6].

One of the implications of food insecurity is adverse health outcomes, with the condition a leading cause of health and nutritional problems globally. According to the Lancet’s 2019 Global Burden of Disease Report, child and maternal malnutrition was the leading Level 2 risk factor for disability-adjusted life years (DALYs) globally in 2019, accounting for 295 million DALYs (11.6% of global DALYs) [14]. Moreover, SSA and south Asia remain particularly vulnerable to malnutrition in general and undernutrition in particular and its health consequences [15].

Many developing country studies have found significant relationships between food insecurity and health (physical and mental). In Ethiopia, for instance, Jebena et al. [16] found, using longitudinal data, that food insecurity is strongly associated with self-rated health. A similar relationship has also been found among Ethiopian adolescents [17]. In Iran, Gholami et al. [18] found a statistically significant association between household food insecurity and mean health-related quality of life as well as the latter’s eight dimensions comprising physical and mental health indicators. Among the Aboriginal population in Canada, food insecurity has been found to be associated with reporting poor general health [19].

Other studies have focused on the relationship between food insecurity and mental health (a specific aspect of general health status). For instance, a systematic review by Lund et al. [20] found a consistent positive relationship between food insecurity and common mental disorders in developing countries. Similarly, in a systematic review of both qualitative and quantitative studies in developing countries, Weaver and Hadley [21], found a positive relationship between food insecurity and mental health outcomes.

Limited evidence exists on the relationship between food insecurity and health in South Africa while some of the evidence is spatially limited. For instance, in Khayelitsha, an urban informal settlement, Case and Deaton [22] found that hunger has a powerful effect on depression while household food expenditure per capita is significantly correlated with self-reported health. In contrast, another study in Khayelitsha reported no relationship between food insecurity (measured as not having food in the house for the next meal) and common mental disorders [20, 23]. However, Havenaar et al. [23] found a positive relationship in Agincourt, another community in South Africa. Similarly, Sorsdahl et al. [24] found that food insufficiency was significantly associated with twelve-month and lifetime DSM-IV outcome.

A few issues are apparent from the above review especially regarding the state of the literature on food insecurity and health outcomes in South Africa. One, there is a paucity of empirical evidence on the issue in general. In addition, most of the literature emanate from community surveys covering a restricted geographical area. Hence, there is a paucity of studies based on nationally representative data.

This paper, therefore, makes an important contribution by examining the relationship between three measures of food insecurity: household hunger, households running out of money to buy food, and the frequency with which households experience hunger on the one hand, and general health, captured by self-assessed health (SAH), on the other. Such a variety of food insecurity measures will assist in ascertaining the robustness of the relationship, if any, while the global nature of the health outcome will provide an indication of the implication of food insecurity for overall health status in contrast to being restricted to a specific indicator of health for which there may or may not be any relationship. Furthermore, examining the relationship over the course of a severe shock like COVID-19 provides an invaluable piece of evidence on how vulnerable the population has become over various stages of the pandemic. Moreover, the longitudinal nature of the underlying dataset provides an opportunity to interrogate important issues like causality (however limited), an advantage over the cross-sectional data available to most of the previous authors who studied the issue in South Africa. Finally, we utilize nationally representative datasets which enable us to make population-wide inferences on the relationship between health and food insecurity in South Africa.

### Theoretical model

Following Blaylock and Blisard [25], we specify a person’s utility as a function of health (H) and food security (FS)^3^:

1 $$U=U\left(FS,H\right), {U}^{^{\prime}}>0, U"<0$$

The individual maximizes utility subject to a health production function^4^:

2 $$H=H\left(FS,G,{X}^{H},M,Y\right)$$

where G denotes the individual’s genetic endowment (e.g. race), X^H^ refers to health-related human capital stocks (e.g. education), while M and Y denote medical inputs (proxied by location, which is indicative of the quality and quantity of medical care and facilities available to an individual) and income/income shocks respectively [25]. An individual therefore combines the inputs in Eq. (2) to produce a given level of health status, where being food secure, favourable genetic endowments/characteristics, higher levels of human capital, better medical inputs and socioeconomic advantage independently and jointly confer health advantages and vice versa (see a more detailed elucidation of the channels of influence in the Methods section below). We assume a unitary model of the household [26], where food security pertains to the household. However, health is measured at the individual level.

## Methods

### Data and variables

We used the National Income Dynamics Study (NIDS)-Coronavirus Rapid Mobile Survey (CRAM) dataset for the analysis [27–30]. NIDS-CRAM is a rapid nationally representative telephonic survey conducted roughly two months apart over the course of the COVID-19 pandemic in South Africa. It is based on the fifth (i.e. final) wave of the adult sample of the NIDS survey. NIDS is the first nationally representative longitudinal survey in South Africa which was conducted between 2008 and 2017 [31].

NIDS-CRAM currently comprises four waves. The first wave was conducted from 7 May to 27 June 2020 while the second wave conducted between 13 July and 13 August 2020. Wave 3 was conducted between 2 November and 13 December 2020, while wave 4 data were collected from 2 February to 10 March 2021.

The survey team employed stratified sampling with batch sampling to select respondents for interview. This entailed providing survey teams with the contact details of sampled respondents in batches of 2,500 randomly drawn from 99 strata. Thus, the sampling rate in each stratum was adjusted as more information became available, allowing for flexibility in the implementation of the survey [32].

Wave 1 of NIDS-CRAM successfully interviewed 7,073 respondents. About 80% of these (5,676 respondents) were successfully re-interviewed in wave 2. Given the 19% attrition rate between wave 1 and wave 2, a top-up sample randomly drawn from the original NIDS wave 5 sample was included in wave 3 resulting in 6,130 observations being successfully interviewed in wave 3. Finally, 5,629 respondents were successfully interviewed in wave 4 [33].

While the descriptive component of the analysis in this paper mostly utilized data from all four waves of the NIDS-CRAM dataset, the main regression analysis (including the descriptive statistics table) only utilized data from wave 1 and wave 4 coinciding with the country’s lockdown levels 5 and adjusted levels 3/1 respectively [8]. This is because the outcome variable was only collected in these two waves.

The outcome variable was an indicator of SAH. Each respondent was asked to rate the present state of their health along a scale with the following options: excellent (1), very good (2), good (3), fair (4) and poor (5). This characterization of health is common in the literature and has been demonstrated to be a significant predictor of mortality in South Africa [34]. In this paper, we dichotomized the variable, grouping respondents with excellent, very good and good together while grouping fair and poor responses together. For convenience, we refer to both groups as the better and worse health groups respectively. Dichotomizing SAH is not uncommon in the literature [9, 31, 35, 36]. We also presented a separate analysis with the original five categories. While SAH is subjective in nature, it has been described as the result of an individual’s rational thought process which takes a holistic view of health and captures even aspects of health that may not be uncovered by more objective measures like clinical tests [37].

The main covariates were three indicators of food insecurity: household hunger, the frequency with which household members experienced hunger, and the respondent’s household running out of money to buy food. Household hunger was obtained from a question about whether anyone in the respondent’s household had gone hungry because there was not enough food in the past 7 days. This was followed by the frequency of hunger episodes, with the following options: never, 1 or 2 days, 3 or 4 days, almost everyday, and everyday. For households lacking money to buy food, respondents were asked whether their household ran out of money to buy food in the preceding month. Thus, food insecurity refers to household welfare and follows the unitary conceptualization of welfare as earlier indicated. Thus, for wave 1 (wave 4), the reference month was around April 2020 (January 2021).

These indicators of food insecurity were complemented by controls drawn from the health literature in a multiple regression context. These were gender, employment status, years of education, type of housing, age, race, negative income shocks, location and household size.

As shown in Eq. (2), health can be produced by food (in)security. Food insecurity (exemplified by hunger and poor diet, for instance) can result in malnutrition and therefore poor health. Moreover, food insecurity may either result in failure to take medical inputs such as drugs or the ineffectiveness of such medical inputs [38]. A typical example is poor adherence to antiretroviral therapy due to hunger [39]. Genetic endowments (proxied by race, for instance) also determine health. In a country like South Africa with a history of racial marginalization, race significantly predicts health given its associated (dis)advantages even after controlling for socioeconomic differences [40]. Similarly, human capital accumulation (through, say, education) is expected to positively affect health, as education may improve health-related decision making as well as result in a more efficient use of health inputs [41, 42]. Also, medical inputs (proxied by location – given its importance for the availability and quality of health services) is important for better health [25]. Furthermore, socioeconomic conditions (proxied by housing conditions, income/income shocks, household size and employment) are generally associated with health, where better housing conditions, higher incomes, employment, and smaller household sizes are generally associated with better health [25]. For instance, an employed household member contributes towards increasing household income, thereby enhancing its food security and ultimately health status, and vice versa. Finally, young people are usually healthier than their older counterparts, an obvious effect of the ageing process [40].

### Analytical methods and models

We employed the linear probability model (LPM) to ascertain the health-food insecurity relationship, with our empirical model taking the following form following the above theoretical model:

$${H}_{i}=\alpha {F}_{i}+{X}^{/}\beta +{\varepsilon }_{i}$$

where H, F, X and $\varepsilon$ are SAH, food insecurity, control variables and an error term respectively, while $\alpha$ and $\beta$ are parameters. Given the five-category nature of the original SAH variable, we also estimated the relationships using ordered logit models. The results (Table 4 in the Appendix) generally led to the similar conclusions as what obtains from our main results. Both the descriptive and econometric estimates were weighted using survey weights designed by the NIDS-CRAM data curators [33].

## Results

### Descriptive analysis

We present the distribution of SAH for wave 1 and wave 4 in Table 1.

**Table 1:** Distribution of health outcomes

Health category	Wave 1 (May/Jun 2020)	Wave 4 (Feb/Mar 2021)
	Percentage	Percentage
Excellent	16.3	16.1
Very good	21.6	24.3
Good	35.4	32.2
Fair	19.4	19.1
Poor	7.3	8.3

Estimates weighted (NIDS-CRAM unbalanced panel)

Table 1 indicates that SAH outcomes were similar between both periods, with about 27% of the population reporting fair/poor health.

**Fig. 1:**
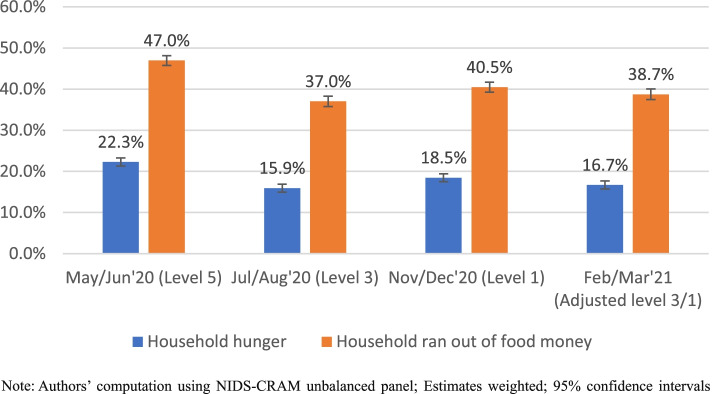
Prevalence of hunger and household running of money to buy food during the coronavirus pandemic in South Africa

Figure 1 tracks the percentage of individuals who reported that their households experienced hunger across the various waves as well as those who indicated that their households ran out of money to buy food.

Figure 1 indicates that 22% of South African adults reported household hunger over the past 7 days in May/June 2020. While this significantly declined in July/August 2020 as the country implemented grant top-ups and eased to level 3 lockdown restrictions, the country recorded a significant 3-percentage point increase in household hunger prevalence in November/December 2020 even though most of the economy had been reopened following a further downgrading of the country’s lockdown restrictions to level 1. Furthermore, the country recorded a non-significant decline in hunger prevalence in February/March 2021. A similar trend obtained regarding households running out of money to buy food across the waves. Considering the latter measure, food insecurity during the pandemic was worse than what one observes from hunger statistics, as 37–47% of adults reported that their households lacked money for food across the waves.

**Fig. 2:**
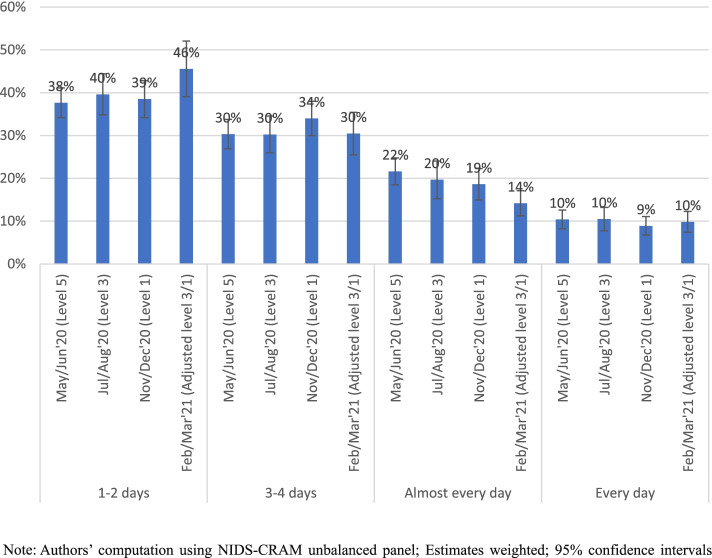
Distribution of hunger rates conditional on household being affected by hunger

Figure 2 reports the distribution of hunger frequencies among those whose households experienced hunger.

Those whose households were affected by hunger for 1 or 2 days in a week dominated, while those whose households experienced hunger everyday were in the minority as expected. However, the levels and stability of everyday hunger experience is worrying given that a tenth of individuals whose households were affected by hunger had to deal with hunger everyday while between a quarter and a third of the hunger-affected population reported experiencing hunger either daily or almost everyday.

**Fig. 3:**
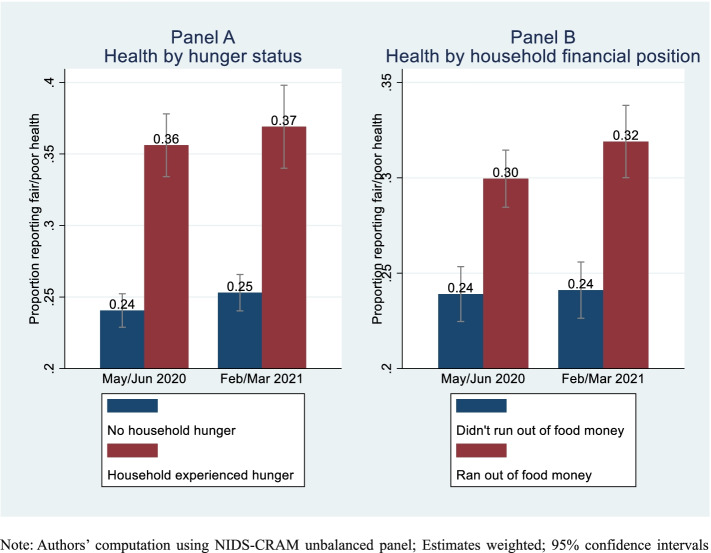
Relationship between health and food insecurity

Figure 3 depicts the relationship between health on the one hand and hunger and lack of money to buy food respectively, on the other.

Both figures provide a clear descriptive evidence of a positive relationship between food insecurity and fair/poor health in both periods. While about a quarter of the adult population with no reported experience of household hunger reported being in fair/poor health (relative to excellent/very good/good health), it ranged between 36–37% for those affected by hunger. Similarly, while a quarter of those whose households ran out of food money reported fair/poor health, it was 30–32% for those whose households ran out of food money.

We present the descriptive statistics of the variables used in the regression analyses in Table 2. The estimation samples for both the descriptive statistics and regression analyses were based on observations with non-missing values in hunger, lack of food money and the regression controls.

**Table 2:** Descriptive statistics

Variable	Wave 1 (Level 5)			Wave 4 (Adjusted level 3/1)		
	(1)	(2)	(3)	(4)	(5)	(6)
	Better health	Worse health	(1)-(2)	Better health	Worse health	(4)-(5)
	Mean/%	Mean/%		Mean/%	Mean/%	
Household experienced hunger in the past 7 days	19.4	29.4	-10.0***	13.7	21.8	-8.1***
**Hunger frequency over the past 7 days**^**a**^						
No one in household experienced hunger	81.9	71.8	10.2***	86.5	78.5	8.0***
Household experienced hunger 1–2 days	6.7	11.2	-4.5***	6.7	9.4	-2.7*
Household experienced hunger 3–4 days	6.0	7.8	-1.9*	3.7	6.9	-3.2***
Household experienced hunger almost every day	3.8	5.5	-1.7**	1.9	2.8	-0.8
Household experienced hunger every day	1.6	3.7	-2.1***	1.2	2.4	-1.2*
Household ran out of money to buy food last month	44.7	51.4	-6.7***	36.0	44.9	-8.9***
Main income source lost/declined^b^	38.8	42.6	-3.8*	17.5	25.8	-8.4***
Male	46.8	48.0	-1.1	47.6	43.5	4.1*
Not employed	53.2	60.9	-7.7***	48.4	57.1	-8.7***
Lives in a house or flat (base = traditional/informal/other type of house)	80.6	76.7	3.9**	81.6	73.6	8.1***
**Race**						
African	73.5	90.3	-16.8***	71.7	92.9	-21.2***
Coloured	11.8	4.8	6.9***	12.8	4.0	8.8***
Asian/Indian	2.9	1.6	1.3*	3.4	1.2	2.1**
White	11.8	3.2	8.6***	12.1	1.9	10.2***
**Location**						
Traditional location	13.0	16.5	-3.6**	19.0	27.4	-8.4***
Urban location	83.5	79.6	3.9**	79.3	69.1	10.1***
Farm location	3.6	3.9	-0.3	1.7	3.4	-1.7**
Years of schooling	11.4	10.6	0.9	11.6	10.4	1.3***
Age (in years)	39.9	40.9	-1.1	41.3	42.4	-1.1
Household size	4.9	5.2	-0.3*	4.6	5.3	-0.7***
**Province**						
Western Cape	13.0	7.8	5.2***	14.6	4.9	9.7***
Eastern Cape	11.2	11.7	-0.5	13.3	9.0	4.3***
Northern Cape	2.8	3.2	-0.4	3.2	2.9	0.3
Free State	6.2	4.8	1.4*	6.8	3.7	3.1***
Kwazulu-Natal	16.2	22.1	-5.9***	12.9	25.2	-12.3***
North-West	3.7	8.8	-5.1***	4.1	9.5	-5.4***
Gauteng	28.7	22.7	6.0**	27.4	26.4	1.0
Mpumalanga	8.5	9.0	-0.5	7.6	10.8	-3.2**
Limpopo	9.7	9.9	-0.1	10.2	7.6	2.6*
Number of observations	4653	1933		3400	1493	

Estimates weighted by the wave-specific weights; *. **. *** indicate statistical significance at 10%, 5% and 1% levels of significance respectively;

^a^N: Wave 1 = 6,451, Wave 4 = 4,875;

^b^Wave 1: whether household lost its main source of income, wave 4: whether household’s main source of income decreased (relative to increased or remained unchanged)

Table 2 highlights the statistically significant differences between the population in the better health group and those in the worse health group for most of the variables included in the analysis. For instance, the prevalence of hunger in the better health group was 10 (8) percentage points lower than in the worse health group in wave 1 (wave 4). Also, the proportion of the worse health group who reported somebody in their household going hungry everyday in wave 1 – not conditional on experiencing hunger –was at least double what obtained in the better health group in both waves, with the differences statistically significant. Similarly, the worse health group reported significantly higher prevalence of running out of money to buy food, with a 7–9 percentage point difference in both waves. Thus, for all the food insecurity indicators, there was descriptive evidence of worse outcomes among those in worse health than those in better health. Furthermore, the better health group was significantly better educated than those in the worse health group in wave 4.

### Econometric analysis

Table 3 provides a set of regression analyses of the relationship between SAH and food insecurity. For each wave, we regressed SAH on individual food insecurity indicators and the controls while finally including both hunger and lack of food money in the final specification (columns 4 and 8).

**Table 3:** The relationship between self-assessed health and food insecurity in South Africa

	**(1)**	**(2)**	**(3)**	**(4)**	**(5)**	**(6)**	**(7)**	**(8)**
	Wave 1 (Level 5)				Wave 4 (Adjusted level 3/1)			
Household experienced hunger in past 7 days	0.067***			0.066***	0.040			0.036
	(0.021)			(0.023)	(0.032)			(0.035)
**Hunger frequency past 7 days (base = no hunger)**								
1–2 days		0.098***				0.027		
		(0.035)				(0.045)		
3–4 days		0.035				0.061		
		(0.034)				(0.045)		
Almost every day		0.061				0.023		
		(0.037)				(0.057)		
Every day		0.148**				0.063		
		(0.057)				(0.069)		
Household ran out of money to buy food in the past month			0.020	0.001			0.020	0.008
			(0.018)	(0.019)			(0.021)	(0.022)
Male	0.015	0.018	0.016	0.015	-0.014	-0.014	-0.014	-0.014
	(0.017)	(0.017)	(0.017)	(0.017)	(0.018)	(0.018)	(0.018)	(0.018)
Not employed	0.037**	0.035*	0.039**	0.037**	0.017	0.018	0.017	0.017
	(0.018)	(0.018)	(0.018)	(0.018)	(0.020)	(0.020)	(0.019)	(0.020)
Years of schooling	-0.003	-0.003	-0.003	-0.003	-0.007**	-0.007**	-0.007**	-0.007**
	(0.003)	(0.003)	(0.003)	(0.003)	(0.003)	(0.003)	(0.003)	(0.003)
Lives in a house/flat (base = lives in a traditional/informal/other type house)	0.002	0.003	-0.001	0.002	-0.049*	-0.050*	-0.050*	-0.049*
	(0.020)	(0.020)	(0.020)	(0.020)	(0.027)	(0.027)	(0.027)	(0.027)
Age (years)	0.002**	0.001**	0.001**	0.002**	0.002***	0.002***	0.002***	0.002***
	(0.001)	(0.001)	(0.001)	(0.001)	(0.001)	(0.001)	(0.001)	(0.001)
**Race (base = African)**								
Coloured	-0.185***	-0.183***	-0.192***	-0.185***	-0.165***	-0.167***	-0.169***	-0.166***
	(0.032)	(0.032)	(0.033)	(0.033)	(0.045)	(0.044)	(0.045)	(0.046)
Asian/Indian	-0.170***	-0.172***	-0.181***	-0.170***	-0.261***	-0.261***	-0.265***	-0.261***
	(0.062)	(0.064)	(0.062)	(0.062)	(0.062)	(0.062)	(0.062)	(0.062)
White	-0.205***	-0.202***	-0.209***	-0.205***	-0.218***	-0.219***	-0.219***	-0.217***
	(0.030)	(0.030)	(0.031)	(0.030)	(0.033)	(0.032)	(0.033)	(0.033)
**Location (base = Traditional)**								
Urban	0.013	0.016	0.009	0.013	-0.033	-0.032	-0.033	-0.033
	(0.026)	(0.026)	(0.026)	(0.026)	(0.024)	(0.024)	(0.024)	(0.024)
Farm	0.007	0.012	0.004	0.007	0.093	0.095	0.091	0.093
	(0.044)	(0.043)	(0.044)	(0.044)	(0.066)	(0.067)	(0.067)	(0.067)
Main income source lost/declined^a^	0.001	0.000	0.004	0.001	0.074***	0.074***	0.075***	0.074***
	(0.017)	(0.017)	(0.017)	(0.017)	(0.026)	(0.026)	(0.026)	(0.026)
Household size	-0.001	-0.001	-0.001	-0.001	0.006*	0.006*	0.006*	0.006*
	(0.003)	(0.003)	(0.003)	(0.003)	(0.003)	(0.003)	(0.003)	(0.003)
**Province (base = Western Cape)**								
Eastern Cape	-0.021	-0.017	-0.023	-0.021	-0.046	-0.046	-0.045	-0.045
	(0.038)	(0.037)	(0.038)	(0.038)	(0.043)	(0.043)	(0.043)	(0.044)
Northern Cape	0.060	0.058	0.064	0.060	0.092*	0.091*	0.094*	0.092*
	(0.040)	(0.041)	(0.040)	(0.041)	(0.053)	(0.053)	(0.053)	(0.053)
Free State	-0.090**	-0.082**	-0.090**	-0.090**	-0.060	-0.061	-0.058	-0.060
	(0.038)	(0.037)	(0.038)	(0.038)	(0.050)	(0.050)	(0.050)	(0.050)
Kwazulu-Natal	0.037	0.051	0.036	0.037	0.184***	0.181***	0.185***	0.184***
	(0.035)	(0.034)	(0.035)	(0.035)	(0.049)	(0.048)	(0.048)	(0.049)
North-West	0.154***	0.152***	0.155***	0.154***	0.211***	0.211***	0.212***	0.211***
	(0.043)	(0.042)	(0.044)	(0.043)	(0.057)	(0.057)	(0.058)	(0.058)
Gauteng	-0.036	-0.034	-0.040	-0.036	0.077*	0.075*	0.078*	0.077*
	(0.033)	(0.032)	(0.033)	(0.033)	(0.042)	(0.042)	(0.042)	(0.043)
Mpumalanga	-0.015	-0.008	-0.018	-0.015	0.080	0.076	0.082	0.081
	(0.040)	(0.040)	(0.041)	(0.040)	(0.050)	(0.050)	(0.050)	(0.051)
Limpopo	-0.037	-0.029	-0.044	-0.037	-0.059	-0.059	-0.061	-0.059
	(0.041)	(0.040)	(0.042)	(0.041)	(0.056)	(0.056)	(0.056)	(0.056)
Constant	0.231***	0.230***	0.252***	0.231***	0.263***	0.264***	0.262***	0.260***
	(0.064)	(0.064)	(0.067)	(0.065)	(0.079)	(0.079)	(0.078)	(0.079)
Observations	6,586	6,451	6,586	6,586	4,893	4,875	4,893	4,893
R-squared	0.059	0.063	0.056	0.059	0.119	0.119	0.118	0.119

All models are LPM; Outcome is SAH, i.e. fair/poor health (base = excellent/very good/good health); Estimates weighted; *, **, *** indicate statistical significance at 10%, 5% and 1% levels of significance respectively; Standard errors in parentheses; ^a^ Wave 1: whether household lost its main source of income, Wave 4: whether household’s main source of income decreased (relative to increased or remained unchanged)

**Table 4:** The relationship between health and food insecurity (ordered logit)

	**(1)**	**(2)**	**(3)**	**(4)**	**(5)**	**(6)**	**(7)**	**(8)**
	Wave 1 (Level 5)				Wave 4 (Adjusted level 3/1)			
Household experienced hunger in past 7 days	0.267***			0.222**	0.213			0.199
	(0.094)			(0.103)	(0.165)			(0.173)
**Hunger frequency past 7 days (base = no hunger)**								
1–2 days		0.283*				0.117		
		(0.155)				(0.266)		
3–4 days		0.139				0.446*		
		(0.158)				(0.240)		
Almost every day		0.332*				-0.053		
		(0.171)				(0.316)		
Every day		0.652***				0.398*		
		(0.243)				(0.237)		
Household ran out of money to buy food in the past month			0.179**	0.116			0.088	0.025
			(0.078)	(0.086)			(0.098)	(0.099)
Male	-0.077	-0.071	-0.067	-0.071	-0.176*	-0.179*	-0.176*	-0.175*
	(0.074)	(0.075)	(0.074)	(0.075)	(0.095)	(0.094)	(0.095)	(0.095)
Not employed	0.103	0.102	0.106	0.097	0.095	0.097	0.100	0.094
	(0.079)	(0.079)	(0.078)	(0.079)	(0.088)	(0.088)	(0.087)	(0.088)
Years of schooling	-0.035***	-0.036***	-0.035***	-0.034***	-0.042***	-0.042***	-0.042***	-0.042***
	(0.011)	(0.011)	(0.011)	(0.011)	(0.013)	(0.013)	(0.013)	(0.013)
Lives in a house/flat (base = lives in a traditional/informal/other type house)	-0.061	-0.062	-0.067	-0.056	-0.048	-0.043	-0.052	-0.046
	(0.098)	(0.100)	(0.101)	(0.100)	(0.127)	(0.130)	(0.126)	(0.127)
Age (years)	0.010***	0.010***	0.010***	0.010***	0.018***	0.018***	0.018***	0.018***
	(0.003)	(0.003)	(0.003)	(0.003)	(0.003)	(0.003)	(0.003)	(0.003)
**Race (base = African)**								
Coloured	-1.388***	-1.377***	-1.426***	-1.399***	-1.506***	-1.520***	-1.522***	-1.509***
	(0.200)	(0.201)	(0.198)	(0.199)	(0.296)	(0.297)	(0.291)	(0.296)
Asian/Indian	-1.017***	-1.008***	-1.066***	-1.029***	-1.526***	-1.525***	-1.548***	-1.526***
	(0.367)	(0.371)	(0.365)	(0.366)	(0.370)	(0.369)	(0.368)	(0.369)
White	-1.469***	-1.460***	-1.475***	-1.460***	-1.193***	-1.200***	-1.200***	-1.192***
	(0.146)	(0.146)	(0.147)	(0.146)	(0.172)	(0.171)	(0.172)	(0.172)
**Location (base = Traditional)**								
Urban	-0.047	-0.022	-0.064	-0.050	-0.060	-0.054	-0.059	-0.060
	(0.101)	(0.105)	(0.101)	(0.102)	(0.104)	(0.104)	(0.104)	(0.104)
Farm	-0.095	-0.074	-0.109	-0.100	0.302	0.308	0.282	0.301
	(0.182)	(0.187)	(0.185)	(0.183)	(0.267)	(0.269)	(0.265)	(0.267)
Main income source lost/declined^a^	0.046	0.037	0.026	0.016	0.338***	0.340***	0.347***	0.337***
	(0.074)	(0.074)	(0.074)	(0.073)	(0.104)	(0.105)	(0.103)	(0.104)
Household size	-0.014	-0.013	-0.016	-0.015	0.020	0.020	0.022	0.020
	(0.013)	(0.013)	(0.013)	(0.013)	(0.014)	(0.014)	(0.015)	(0.014)
**Province (base = Western Cape)**								
Eastern Cape	-0.116	-0.103	-0.115	-0.111	0.098	0.091	0.103	0.099
	(0.178)	(0.178)	(0.179)	(0.178)	(0.242)	(0.241)	(0.240)	(0.242)
Northern Cape	-0.109	-0.115	-0.088	-0.102	0.539**	0.524**	0.544**	0.539**
	(0.205)	(0.206)	(0.206)	(0.206)	(0.232)	(0.230)	(0.231)	(0.232)
Free State	-0.447**	-0.398**	-0.446**	-0.448**	0.008	-0.011	0.015	0.010
	(0.177)	(0.172)	(0.179)	(0.177)	(0.257)	(0.256)	(0.255)	(0.257)
Kwazulu-Natal	0.073	0.093	0.073	0.076	0.862***	0.838***	0.867***	0.862***
	(0.163)	(0.163)	(0.164)	(0.163)	(0.260)	(0.262)	(0.258)	(0.261)
North-West	0.196	0.187	0.193	0.191	0.688**	0.679**	0.691**	0.688**
	(0.203)	(0.203)	(0.206)	(0.203)	(0.286)	(0.287)	(0.284)	(0.286)
Gauteng	-0.236	-0.225	-0.249	-0.235	0.267	0.249	0.270	0.268
	(0.163)	(0.162)	(0.164)	(0.164)	(0.245)	(0.245)	(0.242)	(0.245)
Mpumalanga	-0.045	-0.034	-0.057	-0.045	0.367	0.334	0.373	0.368
	(0.174)	(0.175)	(0.175)	(0.174)	(0.265)	(0.267)	(0.262)	(0.266)
Limpopo	-0.138	-0.106	-0.166	-0.140	-0.387	-0.396	-0.395	-0.387
	(0.206)	(0.207)	(0.205)	(0.206)	(0.284)	(0.285)	(0.283)	(0.285)
Cutoff 1	-2.234***	-2.201***	-2.243***	-2.178***	-1.517***	-1.515***	-1.513***	-1.506***
	(0.298)	(0.302)	(0.300)	(0.301)	(0.377)	(0.375)	(0.372)	(0.373)
Cutoff 2	-0.959***	-0.929***	-0.968***	-0.903***	-0.076	-0.077	-0.074	-0.065
	(0.305)	(0.309)	(0.308)	(0.308)	(0.376)	(0.374)	(0.370)	(0.372)
Cutoff 3	0.672**	0.692**	0.662**	0.729**	1.478***	1.478***	1.479***	1.488***
	(0.301)	(0.305)	(0.305)	(0.305)	(0.379)	(0.378)	(0.373)	(0.376)
Cutoff 4	2.289***	2.322***	2.275***	2.346***	3.041***	3.039***	3.041***	3.052***
	(0.315)	(0.317)	(0.317)	(0.317)	(0.393)	(0.391)	(0.385)	(0.389)
Observations	6,586	6,451	6,586	6,586	4,893	4,875	4,893	4,893
*P*-value	0	0	0	0	0	0	0	0

Outcome is five-category SAH, with higher values indicating worsening health; Estimates weighted; *, **, *** indicate statistical significance at 10%, 5% and 1% levels of significance respectively; Standard errors in parentheses; ^a^ Wave 1: whether household lost its main source of income, Wave 4: whether household’s main source of income decreased (relative to increased or remained unchanged)

**Table 5:** Relationship between lagged food insecurity and health

	**(1)**	**(2)**	**(3)**	**(4)**
Lagged household hunger	0.057**			0.036
	(0.025)			(0.027)
Lagged no food money in household		0.060***		0.050**
		(0.021)		(0.023)
**Lagged hunger frequency past 7 days (base = no hunger)**				
1–2 days			0.072*	
			(0.039)	
3–4 days			0.047	
			(0.042)	
Almost every day			0.025	
			(0.046)	
Every day			0.067	
			(0.079)	
Male	-0.014	-0.011	-0.014	-0.010
	(0.021)	(0.021)	(0.021)	(0.021)
Not employed	0.023	0.023	0.024	0.024
	(0.022)	(0.022)	(0.022)	(0.022)
Years of schooling	-0.006*	-0.006*	-0.006*	-0.006*
	(0.003)	(0.003)	(0.003)	(0.003)
Lives in a house/flat (base = lives in a traditional/informal/other type house)	-0.036	-0.028	-0.040	-0.027
	(0.026)	(0.027)	(0.027)	(0.027)
Age (years)	0.002***	0.002***	0.002**	0.002***
	(0.001)	(0.001)	(0.001)	(0.001)
**Race (base = African)**				
Coloured	-0.148***	-0.160***	-0.146***	-0.154***
	(0.037)	(0.038)	(0.037)	(0.037)
Asian/Indian	-0.235***	-0.254***	-0.234***	-0.244***
	(0.086)	(0.084)	(0.087)	(0.084)
White	-0.220***	-0.217***	-0.217***	-0.214***
	(0.034)	(0.035)	(0.034)	(0.035)
**Location (base = Traditional)**				
Urban	-0.018	-0.015	-0.022	-0.016
	(0.027)	(0.027)	(0.028)	(0.027)
Farm	0.096	0.091	0.093	0.098
	(0.074)	(0.070)	(0.074)	(0.072)
Main income source lost/declined	0.075***	0.068**	0.078***	0.070**
	(0.028)	(0.028)	(0.028)	(0.028)
Household size	0.005	0.005	0.005	0.004
	(0.004)	(0.004)	(0.004)	(0.004)
**Province (base = Western Cape)**				
Eastern Cape	-0.043	-0.041	-0.040	-0.042
	(0.038)	(0.039)	(0.038)	(0.038)
Northern Cape	0.062	0.069	0.062	0.067
	(0.046)	(0.047)	(0.046)	(0.046)
Free State	-0.057	-0.057	-0.052	-0.057
	(0.041)	(0.041)	(0.041)	(0.041)
Kwazulu-Natal	0.181***	0.184***	0.184***	0.179***
	(0.041)	(0.041)	(0.042)	(0.041)
North-West	0.222***	0.222***	0.219***	0.222***
	(0.055)	(0.056)	(0.055)	(0.056)
Gauteng	0.085**	0.081**	0.082**	0.082**
	(0.036)	(0.036)	(0.036)	(0.036)
Mpumalanga	0.079*	0.076*	0.070	0.077*
	(0.044)	(0.044)	(0.044)	(0.044)
Limpopo	-0.055	-0.061	-0.058	-0.058
	(0.049)	(0.049)	(0.049)	(0.049)
Constant	0.220***	0.201**	0.230***	0.190**
	(0.078)	(0.079)	(0.077)	(0.079)
Observations	4,169	4,168	4,081	4,150
R-squared	0.114	0.117	0.113	0.117

All models are LPM; Outcome is SAH, i.e. fair/poor health (base = excellent/very good/good health); Estimates weighted; *, **, *** indicate statistical significance at 10%, 5% and 1% levels of significance respectively; Standard errors in parentheses

Table 3 indicates that household hunger was positively and significantly associated with worse health outcomes in wave 1, with one’s household experiencing hunger in the past seven days associated with a 7-percentage point higher probability of reporting worse health compared to not experiencing hunger. It however lost statistical significance in wave 4 even though the relationship remained numerically nontrivial. Expectedly, the frequency with which households experienced hunger had important implications for health especially in wave 1. Those who reported their households experiencing hunger for 1–2 days and everyday were significantly more likely to report worse health in wave 1 compared to those who did not experience hunger.

The control variables generally conformed to expectations. Non-employment, age, negative income shocks, a larger household size, and residence in the Northern Cape, Kwazulu-Natal, North-West and Gauteng (relative to the Western Cape, arguably the most industrialized province) were significantly associated with poor health in at least one wave. Conversely, education, being non-African, living in better housing and residence in Free State were negatively associated with worse health outcomes.

To partially address the possible reverse causality between SAH and food insecurity, we regressed SAH on lagged indicators of food insecurity (and contemporaneous controls) – see Table 5 in the Appendix. The results showed positive and statistically significant relationships for specifications with separate hunger and lack of food money (6-percentage point effects each). Even when both indicators were entered in the same regression, both coefficients remained numerically nontrivial (4 and 5-percentage point effects respectively), with the coefficient for lack of food money retaining statistical significance (*p* < 0.05).

## Discussion

This paper shows that many South Africans reported adverse health outcomes, with more than a quarter reporting being in fair/poor health in May/June 2020 and February/March 2021. Moreover, food insecurity has remained both substantial and chronic. This is worrying and lays bare an economy that was already in dire straits with worryingly high food insecurity and economic decline prior to the pandemic [13, 43]. Thus, an already bad situation was compounded by a pandemic that has severely weakened the country in terms of severe job losses (that are yet to fully recover), income loss and the associated anxiety [10, 12].

It is important to note that the significant decline in hunger (between May/June and July/August 2020) coincided with the period when grants were increased as part of the pandemic response. The subsequent uptick and insignificant decline in hunger in the face of a greater relaxation of lockdown restrictions is consistent with a high unemployment economy where a simple relaxation of lockdown restrictions is not enough to significantly enhance people’s livelihoods. Moreover, it was not only that hunger prevalence was high. Among those households that experienced hunger, about 10% across the waves indicated that it was an everyday experience while between a quarter and a third of the hunger-affected population indicated a hunger experience that occurred either daily or almost everyday.^5^

In both the descriptive and regression analyses, there was a numerically nontrivial and statistically significant positive relationship between hunger and worse health outcomes. In a population sense, this is significant given the high prevalence of fair/poor health– about 27%. This concurs with Willows et al. [19] who found that food insecurity was positively and significantly associated with general self-reported poor health among the Canadian Aboriginal community.

In contrast to hunger, the statistically significant bivariate relationship between health and running out of money to buy food (Panel B of Fig. 3) was lost when relevant confounding factors were controlled for in a contemporaneous sense. However, exploiting the longitudinal nature of the data, prior experience of lacking money to purchase food provided a stronger (and significant) adverse effect on health than what obtained contemporaneously. This concurs with some of the results found by Sharkey et al. [36] in the USA. Their food insecurity measure, while similar to ours, was significantly associated with self-reporting fair/poor health in the general model (as well as reporting poor physical health and frequent mental distress). Similarly, among low-income Americans, food insecurity (including a measure of inadequate resources to purchase food) was significantly associated with potentially health-endangering behaviour like postponing medical care [44].

We also found that relative to not experiencing hunger, those who experienced hunger for varying lengths of time in the past week were more likely to report worse health, with hunger experience for 1–2 days and everyday statistically significant. This finding is similar to another study which found that repeated hunger episodes were very toxic for children’s health and that multiple episodes of hunger (relative to never experiencing hunger) were associated with higher likelihood of asthma and chronic conditions among children [45, 46]. However, we did not find comparable studies which analysed adults.

Regarding the controls, numerous studies corroborate our findings. For instance, better educational attainment is known to be negatively (positively) associated with worse (better) health [47, 48]. However, Case and Deaton [22] found no evidence that better educated people suffer less from poor health than their less educated counterparts in South Africa and India. The latter may be due to the restricted nature of their study as it was based on a low-income township in South Africa and a city in India. Conversely, older people have poorer health outcomes, a consequence of the ageing process [47, 49]. Moreover, as found above, poor housing conditions have been associated with poor health outcomes especially through overcrowding, poor sanitation and other environmental hazards [50].

A limitation of this study is the fact that while health was measured at the individual level, food insecurity was measured at the household level. While this concurs with the unitary conceptualization of the household, subsequent work on intra-household bargaining suggest that access to household resources may differ across household members [51, 52]. Therefore, it is possible for an individual belonging to a so-called food secure household to be food insecure and vice versa. Perhaps, this explains some of the statistically insignificant relationships obtained in this paper. In such a scenario, our findings may be viewed as lower bounds of the impact of food insecurity on health.

## Conclusion

This paper has investigated an important issue – whether food insecurity is significantly related to health outcomes in South Africa during the COVID-19 pandemic. This is especially important given widespread food insecurity during the pandemic as well as a large proportion of the population self-reporting worse health outcomes. It is worrying that rather than witnessing a substantial decline in food insecurity in recent months with the further re-opening of the economy, food insecurity remains stubborn at very high levels. The only significant reduction occurred during the period when grant top-ups were disbursed. Individuals whose households experience hunger have a 7-percentage point higher probability of reporting worse health outcomes relative to those whose households do not experience hunger. Those whose households experience hunger everyday have a 15-percentage point higher probability of reporting worse health than those unaffected by hunger.

The foregoing highlights the urgent need to address food insecurity in the country especially as some of the pandemic-induced palliatives are being removed. There is far too much hunger in the country especially as many jobs are yet to recover, if not permanently gone. This study highlights the wider implication of food insecurity, especially its extreme manifestation in hunger, namely that food insecurity has adverse health implications. It is hoped that policy makers and implementers as well as the private sector and non-profit organizations will redouble efforts to significantly alleviate the scourge of food insecurity in South Africa.

## Data Availability

The datasets generated and/or analysed during the current study are available in the Data first repository, https://www.datafirst.uct.ac.za/dataportal/index.php/catalog/817.

## Appendix

Tables 4 and 5

## Abbreviations

COVID-19: The 2019 coronavirus disease
DALY: Disability-Adjusted Life Year
FAO: Food and Agricultural Organization
NIDS-CRAM: National Income Dynamics Study-Coronavirus Rapid Mobile Survey
SAH: Self-Assessed Health
SSA: Sub-Saharan Africa
USD: United States Dollar
ZAR: South African Rand

## Footnotes

1 http://www.fao.org/3/ca9692en/online/ca9692en.html#chapter-executive_summary

2 https://www.ipsos.com/en-za/almost-half-south-african-households-go-hungry-due-covid-19

3 While there are obviously other determinants of utility, we restrict the discussion here to these two arguments for simplicity.

4 One may think of other constraints, such as a food security production function. For simplicity, we only consider the constraint specified Eq. (2).

5 About 7% (4%) of the general population experienced hunger almost everyday or everyday in wave 1 (wave 4).

## References

[1] FAO (2001). The State of Food and Agriculture 2001.

[2] World Bank. Food security and COVID-19 2021 [cited 2021 17 April]. Available from: https://www.worldbank.org/en/topic/agriculture/brief/food-security-and-covid-19.

[3] United Nations. Policy brief: The impact of COVID-19 on food security and nutrition. The United Nations; 2020. Available at: https://unsdg.un.org/resources/policy-brief-impact-covid-19-food-security-and-nutrition.

[4] Statistics South Africa. Towards measuring the extent of food security in South Africa: An examination of hunger and food inadequacy. Pretoria: Statistics South Africa; 2019. Report No.: 03-00-14. Available at: http://www.statssa.gov.za/publications/03-00-14/03-00-142017.pdf.

[5] Wills G, Patel L, Van der Berg S, Mpeta B. Household resource flows and food poverty during South Africa’s lockdown: Short-term policy implications for three channels of social protection. 2020. Wave 1 NIDS-CRAm Working Paper, No. 12. Available at: https://cramsurvey.org/wp-content/uploads/2020/07/Wills-household-resource-flows-and-food-poverty-during-South-Africa%E2%80%99s-lockdown-2.pdf.

[6] Van der Berg S, Patel L, Bridgman G. Hunger in South Africa during 2020: Results from Wave 3 of NIDS-CRAM. 2021. Wave 3 NIDS-CRAM Working Paper, No. 10. Available at: https://cramsurvey.org/wp-content/uploads/2021/02/10.-Van-der-Berg-S.-Patel-L.-Bridgman-G.-2021-Hunger-in-South-Africa-during-2020-Results-from-Wave-3-of-NIDS-CRAM-1.pdf.

[7] Kohler T, Bhorat H. Social assistance during South Africa’s national lockdown: Examining the COVID-19 grant, changes to the Child Support Grant, and post-October policy options. 2020. Wave 2 NIDS-CRAM Working Paper, No. 9. Available at: https://cramsurvey.org/wp-content/uploads/2020/09/9.-Ko%CC%88hler-T.-_-Bhorat-H.-2020-Social-assistance-during-South-Africa%E2%80%99s-national-lockdown-Examining-the-COVID-19-grant-changes-to-the-Child-Support-Grant-and-post-October-policy-options.pdf.

[8] South African Government. COVID-19 / Novel Coronavirus 2021. 2021. [Accessed 19 April 2021]. Available from: https://www.gov.za/Coronavirus#.

[9] Nwosu CO, Oyenubi A (2021). Income-related health inequalities associated with the coronavirus pandemic in South Africa: A decomposition analysis. Int J Equity Health.

[10] Statistics South Africa (2020). usiness impact survey of the COVID-19 pandemic in South Africa.

[11] Spaull N, The NIDS-CRAM Team. Overview and findings: NIDS-CRAM synthesis report wave 1. 2020. Wave 1 NIDS-CRAM Working Paper, No. 1. Available at: https://cramsurvey.org/wp-content/uploads/2020/07/Spaull-et-al.-NIDS-CRAM-Wave-1-Synthesis-Report-Overview-and-Findings-1.pdf.

[12] Statistics South Africa. Quarterly labour force survey, Quarter 1, 2021. Pretoria: Statistics South Africa; 2021. Report No.: P0211. Available at: https://www.statssa.gov.za/publications/P0211/P02111stQuarter2021.pdf.

[13] NDoH, Statistics South Africa, SAMRC, ICF. South Africa Demographic and Health Survey 2016 key findings. Pretoria, South Africa and Rockville, Maryland: NDoH, Stats SA, SAMRC, and ICF; 2018. Available at: https://dhsprogram.com/pubs/pdf/SR248/SR248.pdf.

[14] Murray CJ, Aravkin AY, Zheng P, Abbafati C, Abbas KM, Abbasi-Kangevari M (2020). Global burden of 87 risk factors in 204 countries and territories, 1990–2019: a systematic analysis for the Global Burden of Disease Study 2019. The Lancet.

[15] Müller O, Krawinkel M (2005). Malnutrition and health in developing countries. CMAJ.

[16] Jebena MG, Lindstrom D, Lachat C, Belachew T, Kolsteren P (2017). The effect of food insecurity on health status of adolescents in Ethiopia: longitudinal study. BMC Public Health.

[17] Hadley C, Lindstrom D, Tessema F, Belachew T (2008). Gender bias in the food insecurity experience of Ethiopian adolescents. Soc Sci Med.

[18] Gholami A, Khazaee-Pool M, Rezaee N, Amirkalali B, Ghahremanlo A, Moradpour F (2017). Household food insecurity is associated with health-related quality of life in rural type 2 diabetic patients. Arch Iran Med.

[19] Willows N, Veugelers P, Raine K, Kuhle S (2011). Associations between household food insecurity and health outcomes in the Aboriginal population (excluding reserves). Health Rep.

[20] Lund C, Breen A, Flisher AJ, Kakuma R, Corrigall J, Joska JA (2010). Poverty and common mental disorders in low and middle income countries: A systematic review. Soc Sci Med.

[21] Weaver LJ, Hadley C (2009). Moving beyond hunger and nutrition: a systematic review of the evidence linking food insecurity and mental health in developing countries. Ecol Food Nutr.

[22] Case A, Deaton A (2005). Health and wealth among the poor: India and South Africa compared. American Economic Review.

[23] Havenaar JM, Geerlings MI, Vivian L, Collinson M, Robertson B (2008). Common mental health problems in historically disadvantaged urban and rural communities in South Africa: prevalence and risk factors. Soc Psychiatry Psychiatr Epidemiol.

[24] Sorsdahl K, Slopen N, Siefert K, Seedat S, Stein DJ, Williams DR (2011). Household food insufficiency and mental health in South Africa. J Epidemiol Community Health.

[25] Blaylock JR, Blisard WN (1995). Food security and health status in the United States. Appl Econ.

[26] Becker GS (1965). A Theory of the Allocation of Time. Econ J.

[27] NIDS-CRAM. National Income Dynamics Study-Coronavirus Rapid Mobile Survey (NIDS-CRAM) 2020, Wave 2 [dataset]. Version 2.0.0. In: SALDRU [Unit]. Cape Town: DataFirst; 2020. Available at: https://www.datafirst.uct.ac.za/dataportal/index.php/catalog/827.

[28] NIDS-CRAM. National Income Dynamics Study-Coronavirus Rapid Mobile Survey (NIDS-CRAM) 2020, Wave 1 [dataset]. Version 2.0.0. In: SALDRU [Unit]. Cape Town: DataFirst; 2020. Available at: https://www.datafirst.uct.ac.za/dataportal/index.php/catalog/817.

[29] NIDS-CRAM. National Income Dynamics Study-Coronavirus Rapid Mobile Survey (NIDS-CRAM) 2020, Wave 3 [dataset]. Version 2.0.0. In: SALDRU [Unit], editor. Cape Town: DataFirst; 2020. Available at: https://www.datafirst.uct.ac.za/dataportal/index.php/catalog/851.

[30] NIDS-CRAM. National Income Dynamics Study-Coronavirus Rapid Mobile Survey (NIDS-CRAM) 2021, Wave 4 [dataset] Version 2.0.0. In: SALDRU [Unit], editor. Cape Town: Datafirst; 2021. Available at: https://www.datafirst.uct.ac.za/dataportal/index.php/catalog/867.

[31] Nwosu CO, Woolard I (2017). The impact of health on labour force participation in South Africa. S Afr J Econ Hist.

[32] Kerr A, Ardington C, Burger R. Sample design and weighting in the NIDS-CRAM survey. 2020. Wave 1 NIDS-CRAM Working Paper, No. B. Available at: https://cramsurvey.org/wp-content/uploads/2020/07/REPORT-B-CRAM-Sample-Design-and-Weighting-in-the-NIDS-CRAM-survey_v7.pdf.

[33] Ingle K, Brophy T, Daniels R. National Income Dynamics Study - Coronavirus Rapid Mobile Survey (NIDS-CRAM) 2020 Panel User Manual. 2021. Wave 4 NIDS-CRAM Working Paper, No. C1. Available at: https://cramsurvey.org/wp-content/uploads/2021/05/Report-C1-Wave-4-Panel-User-Manual.pdf.

[34] Ardington C, Gasealahwe B (2014). Mortality in South Africa: Socio-economic profile and association with self-reported health. Dev South Afr.

[35] Newbold KB (2005). Self-rated health within the Canadian immigrant population: risk and the healthy immigrant effect. Soc Sci Med.

[36] Sharkey JR, Johnson CM, Dean WR (2011). Relationship of household food insecurity to health-related quality of life in a large sample of rural and urban women. Women Health.

[37] Jylhä M (2009). What is self-rated health and why does it predict mortality? Towards a unified conceptual model. Soc Sci Med.

[38] Hampton T (2007). Food insecurity harms health, well-being of millions in the United States. JAMA.

[39] Weiser SD, Tuller DM, Frongillo EA, Senkungu J, Mukiibi N, Bangsberg DR (2010). Food insecurity as a barrier to sustained antiretroviral therapy adherence in Uganda. PLoS ONE.

[40] Nwosu CO (2021). Childcare and depression during the coronavirus pandemic in South Africa: A gendered analysis. PLoS ONE.

[41] Brunello G, Fort M, Schneeweis N, Winter-Ebmer R (2016). The causal effect of education on health: What is the role of health behaviors?. Health Econ.

[42] Lochner L. Non-production benefits of education: Crime, health, and good citizenship. NBER, WP16722. 2011. Available at: https://www.nber.org/papers/w16722.

[43] Naidoo P. After more than 25 years S. Africa is now junk with Moody’s too 2020 [cited 2021 19 April]. Available from: https://www.bloomberg.com/news/articles/2020-03-27/south-africa-gets-full-house-of-junk-ratings-after-moody-s-cut.

[44] Kushel MB, Gupta R, Gee L, Haas JS (2006). Housing instability and food insecurity as barriers to health care among low-income Americans. J Gen Intern Med.

[45] Ke J, Ford-Jones EL (2015). Food insecurity and hunger: A review of the effects on children's health and behaviour. Paediatr Child Health.

[46] Kirkpatrick SI, McIntyre L, Potestio ML (2010). Child hunger and long-term adverse consequences for health. Arch Pediatr Adolesc Med.

[47] Case A (2004). Does money protect health status? Evidence from South African pensions.

[48] Gravelle H, Sutton M (2009). Income, relative income, and self-reported health in Britain 1979–2000. Health Econ.

[49] Taimela S, Lr E, Malmivaara A, Tiekso J, Sintonen H, Justn S (2007). Self-reported health problems and sickness absence in different age groups predominantly engaged in physical work. Occup Environ Med.

[50] Krieger J, Higgins DL (2002). Housing and health: time again for public health action. Am J Public Health.

[51] Richards E, Theobald S, George A, Kim JC, Rudert C, Jehan K (2013). Going beyond the surface: gendered intra-household bargaining as a social determinant of child health and nutrition in low and middle income countries. Soc Sci Med.

[52] Sen A, Tinker I (1990). Gender and cooperative conflicts. Persistent inequalities: Women and world development.

